# Chronic cerebrospinal venous insufficiency in multiple sclerosis: clinical correlates from a multicentre study

**DOI:** 10.1186/1471-2377-11-132

**Published:** 2011-10-26

**Authors:** Stefano Bastianello, Alfredo Romani, Gisela Viselner, Enrico Colli Tibaldi, Elisabetta Giugni, Marta Altieri, Pietro Cecconi, Laura Mendozzi, Massimiliano Farina, Donatella Mariani, Antonio Galassi, Claudio Quattrini, Marcello Mancini, Vincenzo Bresciamorra, Angela Lagace, Sandy McDonald, Giorgio Bono, Roberto Bergamaschi

**Affiliations:** 1 Department of Public Health and Neurosciences, IRCCS "C. Mondino National Institute of Neurology" Foundation, University of Pavia, Italy; 2 Interdepartmental Research Centre on Multiple Sclerosis, "C. Mondino National Institute of Neurology" Foundation, Pavia Italy; 3 Department of Neurology and Psychiatry and Department of Neurosciences, Sapienza University of Rome, Italy; 4 Multiple Sclerosis Centre, IRCCS Don Gnocchi, Milan, Italy; 5 CCSVI Project, "Policlinico di Monza", University of Milan, Italy; 6 Hospital of Civitanova, Marche, Italy; 7 Department of Neurosciences Federico II, University of Naples, Italy; 8 Barrie Vascular Imaging, Barrie Ontario, Canada; 9 Department of Neurosciences, Ospedale Circolo, University of Varese, Italy

## Abstract

**Background:**

Chronic cerebrospinal venous insufficiency (CCSVI) has recently been reported to be associated with multiple sclerosis (MS). However, its actual prevalence, possible association with specific MS phenotypes, and potential pathophysiological role are debated.

**Method:**

We analysed the clinical data of 710 MS patients attending six centres (five Italian and one Canadian). All were submitted to venous Doppler sonography and diagnosed as having or not having CCSVI according to the criteria of Zamboni et al.

**Results:**

Overall, CCSVI was diagnosed in 86% of the patients, but the frequency varied greatly between the centres. Even greater differences were found when considering singly the five diagnostic criteria proposed by Zamboni et al. Despite these differences, significant associations with clinical data were found, the most striking being age at disease onset (about five years greater in CCSVI-positive patients) and clinical severity (mean EDSS score about one point higher in CCSVI-positive patients). Patients with progressive MS were more likely to have CCSVI than those with relapsing-remitting MS.

**Conclusion:**

The methods for diagnosing CCSVI need to be refined, as the between-centre differences, particularly in single criteria, were excessively high. Despite these discrepancies, the strong associations between CCSVI and MS phenotype suggest that the presence of CCSVI may favour a later development of MS in patients with a lower susceptibility to autoimmune diseases and may increase its severity.

## Background

Multiple sclerosis (MS) is an inflammatory demyelinating disease of the central nervous system with an autoimmune pathogenesis which has been the subject of thousands of clinical and experimental studies [1-3]. Although the causes triggering the autoimmune process are not entirely clear, it is generally accepted that they lie in the interplay between genetic and environmental factors [4-6]

Recently a new "actor" has appeared on the scene, namely, chronic cerebrospinal venous insufficiency (CCSVI), a condition which may be either mostly genetically determined or acquired [7-9]. CCSVI is characterised by multiple stenoses of the extracranial venous draining pathways, i.e. the internal jugular veins and the Azygous veins, which lead to collateral formation, alteration of the blood-brain barrier, and accumulation of iron, which itself could trigger and maintain the autoimmune cascade [7,9,10]. Some well known, but poorly explained, aspects of MS, such as the perivenular location of the lesions, the neurodegeneration and the common presence of iron deposits, seem to fit well with this hypothesis.

There is, however, no general consensus about the actual existence of CCSVI in MS, and its putative pathogenetic role, or about the most efficient tool for diagnosing it [11,12]. Most studies finding significant differences in the prevalence of CCSVI between MS patients and controls used a venous sonographic method in accordance with Zamboni's original report [13]. However, Doepp et al. [14,15], adopting a different sonographic approach, did not find evidence of CCSVI in a group of 56 MS patients. In two further studies, the pathogenetic role of CCSVI was challenged, as the condition was found to be linked only to more advanced disease [16], or to be present only in a very limited proportion (16%) of patients with clinically isolated syndrome [17]. Even less explored is the possibility that MS patients with CCSVI, as opposed to those without, have a characteristic disease 'phenotype'. The finding of phenotypic associations could prove particularly important, steering further pathophysiological research and making it possible to refine a putative 'vascular model' to be integrated with the existing autoimmune one.

In the present research, whose main aims were to assess the variability of CCSVI data between the centres and to seek to identify consistent relations with clinical aspects, we analysed the 'basic' clinical data of MS patients from six MS centres, five Italian and one Canadian. All the patients were submitted to venous echo-colour-Doppler examinations according to the procedure used by Zamboni et al.

## Methods

The centres in which the echo-colour-Doppler examinations were performed are:

Centre 1: Department of Public Health and Neurosciences, IRCCS "C. Mondino National Institute of Neurology" Foundation, University of Pavia, Italy

Centre 2: Barrie Vascular Imaging, Barrie Ontario, Canada

Centre 3: Hospital of Civitanova, Marche, Italy

Centre 4: Department of Neurosciences, Federico II University of Naples, Italy

Centre 5: Multiple Sclerosis Centre, IRCCS Don Gnocchi, Milan, Italy

Centre 6: CCSVI Project, "Policlinico di Monza", Italy

### Patients

We collected data of 805 patients from the above centres, all patients signed a consent form. All had a diagnosis of MS according to McDonald's revised criteria [18] and all were submitted to Doppler sonography in the period between 2 January 2010 and 17 January 2011. No particular inclusion criteria were used, apart from a diagnosis of MS. In this way, we obtained six sets of 'consecutive' assessments of regularly monitored patients who had asked to undergo the procedure.

Of the original 805 patients, 95 were excluded because no data could be obtained regarding their form of MS. Therefore, the final study sample comprised 710 patients (466 females and 244 males).

The following clinical data were considered: sex, age at time of Doppler evaluation, age at disease onset, disease duration at time of Doppler evaluation, and Expanded Disability Status Scale (EDSS) score at time of Doppler evaluation. From these data the Multiple Sclerosis Severity Score (MSSS) score was computed, which is a validated measure of disease severity computed from disability (EDSS) and disease duration data [19].

The echo-colour-Doppler examinations of cerebrospinal venous drainage were performed by nine different trained ultrasound operators using three different ultrasound systems:

- ECD Esaote-Biosound MyLabVinco 25 scanner equipped with a 3.5-10 MHz linear transducer and a 5.0-8.0 MHz microconvex transducer, both for extracranial measurements, and a 2.0-3.3 MHz phased array probe for transcranial analysis (Centres 1, 3,5,6)

- P5 General Electric ultrasound machine equipped with a linear probe 3.5-10 MHz for extracranial examination and a 2.0-3.0 MHz sector probe for transcranial examination (Centre 2);

- Philips iU22 system equipped with a 3.0-9.0 MHz linear wide-band transducer, a 5.0-8.0 MHz microconvex probe, and a 1.0-5.0 MHz phased array transcranial probe (Centre 4).

Each subject was investigated first in supine position and then in sitting position using a tilt chair. All were scanned following the Zamboni protocol for diagnosis of CCSVI, which is based on the detection of five parameters:

1 - Reflux in the internal jugular veins (IJVs) and/or vertebral veins (VVs) in sitting and supine posture.

2 - Reflux in the intracranial veins. Reflux is defined as a reversal of flow direction during the inspiratory and expiratory phase during normal breathing with mouth closed. The transcranial colour-coded duplex sonography (TCCD) studies were carried out using one of two different approaches: the classic transtemporal window or the transcondylar window.

The classic transtemporal window was used by one centre (Centre 4 - 118 MS patients), evaluating flow direction in the Rosenthal vein and transverse sinus, while all the other centres (592 MS patients) used the transcondylar window, which assesses the direction of flow in the cavernous and petrosal sinuses. The transducer was placed at the level of the mandibular condyle, sloping the tail approximately 10 degrees downwards. The insonation depth was set at 11 cm.

3 - B-mode evidence of abnormalities in the IJVs, such as stenoses, malformed valve, annulus, septums, etc.

4 - Flow not Doppler-detectable in IJVs and/or VVs despite numerous deep breaths.

5 - Reverted postural control of the main cerebral venous outflow pathways, detected by measuring the difference in IJV cross-sectional area (CSA) between the supine and upright positions.

ΔCSA in the IJV, obtained by subtracting the CSA measured in sitting position from that measured in supine position, is a positive value in normal subjects. A negative ΔCSA value indicates loss of postural control of the predominant outflow route in the supine position.

This parameter was assessed in B-mode in transversal access, at the J2 point which corresponds to the level of the thyroid gland, and carefully avoiding any compression of the vessel by the probe.

### Statistical analysis

The Kolmogorov-Smirnoff test was used to check the distribution of quantitative variables for deviation from normal. Data were submitted to univariate analysis of variance (UNIANOVA), chi-square statistics (CROSSTABS) and logistic regression analysis using a stepwise forward procedure (NOMREG). The statistical analysis was conducted using SPSSPC+ software.

## Results

Table 1 summarises the clinical data of the MS patients from the six participating centres. All parameters showed highly significant differences (p < 0.001) between the centres, with the exception of disease duration (p = 0.022). The between-centre differences in mean values of quantitative clinical data could not be attributed entirely to different distributions of MS forms, since they were also present when the different MS forms were analysed separately (with the exception of age and EDSS score in the primary progressive group, which did not show significant differences between the centres).

**Table 1:** Clinical data of MS patients from the 6 MS centres; counts, means and (standard deviations)

MS centre	1	2	3	4	5	6	All centres
RR/SP/PP	104/37/7	97/68/44	74/34/8	62/34/22	19/28/2	32/26/12	388/227/95

Age	43(10)	49(11)	43(10)	45(10)	43(10)	44(11)	45(11)

Age at onset	31(10)	36(10)	32(10)	32(8)	29(10)	34(10)	33(10)

Disease duration	12(8)	14(8)	11(8)	13(8)	14(9)	10(6)	12(8)

EDSS	3.5(2.3)	5(2.3)	4.4(2.2)	5.7(1.5)	4.5(1.9)	4.4(2.2)	4.4(2.2)

MSSS	4.1(2.9)	6(2.7)	5.4(2.7)	4.8(1.7)	6.1()	5.3(2.6)	5.3(2.6)

p < 0.001 for all parameters except disease duration (p = 0.022).

RR: relapsing-remitting; SP: secondary progressive; PP: primary progressive

Table 2 shows the proportions of patients, from the six centres, who fulfilled each of the single CCSVI criteria and who had a CCSVI diagnosis (i.e. fulfilled at least two CCSVI criteria). The between-centre differences in the percentages of presence/absence of each of the five CCSVI criteria, as well as of CCSVI diagnosis, were all highly significant (p < 0.001).

**Table 2:** Percentages of positive CCSVI diagnoses and of positive single CCSVI criteria recorded at each MS centre

Doppler abnormality	MS centre						
	**1**	**2**	**3**	**4**	**5**	**6**	**All centres**

Crit 1+ %	55	94	77	60	82	74	75

Crit 2+ %	49	26	46	29	80	91	45

Crit 3+ %	69	86	89	70	84	87	80

Crit 4+ %	21	46	36	37	29	19	34

Crit 5+ %	24	86	18	5	18	23	38

CCSVI+ %	77	96	91	74	82	94	86

p < 0.001 for all parameters

Table 3 shows the distribution of all the possible combinations of 2 or more criteria present in the patients of the study. It appears that the more frequent combinations are: 1+3, 1+2+3, 1+3+5 and 1+3+4+5, which together represent thirty-five percent of cases.

**Table 3:** Number of CCSVI+ patients according to the specific combinantion of Zamboni's Criteria.

Patients with:						
**2 positive Criteria**		Crit1+	Crit2+	Crit3+	Crit4+	
	Crit2+	11				
	Crit3+	75	36			
	Crit4+	10	7	19		
	Crit5+	9	2	4	4	

**3 positive Criteria**		Crit12+	Crit13+	Crit14+	Crit23+	Crit24+
	Crit3+	109				
	Crit4+	4	39		10	
	Crit5+	9	78	5	5	8

**4 positive Criteria**	Crit1234+	Crit1235+	Crit1245+	Crit1345+	Crit2345+	
	22	37	9	63	0	

**5 positive Criteria**	Crit12345+					
	35					

The table 4 shows the distribution of the different criteria in patients with negative CCSVI. In particular, in this group we note that in 23 patients Doppler examination showed the presence of B-mode anomalies (positivity of the third criterion) in the absence of hemodynamic's flow consequences in the vein, mean while 26 patients had hemodynamic's flow anomalies in the jugular veins or vertebral (positivity of the first or fourth criterion), in the absence of anatomical abnormalities. 28 patients (4% of the study population) showed no criterion.

**Table 4:** Clinical characteristics of the 98 CCSVI negative patients.

	Crit1+	Crit2+	Crit3+	Crit4+	Crit5+	anyCrit+
n	20	19	23	6	2	70
EDSS	3,1/3,4	3,5/2,8	3,4/3,5	3,2/4,7	3,3/4,8	3,1/3,5
MSSS	4,0/4,3	4,0/4,1	4,0/4,1	4,0/4,6	4,0/6,4	3,4/4,3
age at onset	28/32	29/31	30/26	29/31	29/26	29/29

n: number of subjects with single positive criteria;

EDSS, MSSS and age at onset: mean values of CCSVI negative patients with single negative/positive Zamboni Criteria. No comparison reaches statistical significance.

Given the high between-centre differences both in the presence of CCSVI (found in 86.2% of patients overall, range: 74%-96%) and in the mean values of the patients' clinical parameters, the presence of possible associations between CCSVI and clinical data was tested by logistic regression analysis using a stepwise forward procedure, entering first the centre defining factor and subsequently the clinical data.

The final model proved to be adequate (chi-square (7 df) = 89.5; p < 0.001). The parameters included in the final model were (in addition to the centre defining factor) EDSS and age at onset. A higher EDSS score and an older age at onset both indicated a higher probability of CCSVI being present.

The parameter estimates are shown in table 5.

**Table 5:** Parameter estimates from the final model of the logistic regression analysis (stepwise forward procedure) performed using CCSVI as the dependent variable.

Parameter Estimates									
**CCSVI^a^**		**B**	**Std Error**	**Wald**	**df**	**Sig**.	**Exp(B)**	**95% Confidence interval for Exp(B)**	
								
								**Lower limit**	**Upper limit**

1	Intercept	1.204	1.153	1.089	1	.297			
	[CENTRE = 1]	-2.199	1.056	4.338	1	.037	.111	.014	.878
	[CENTRE = 2]	.356	1.190	.089	1	.765	1.427	.139	14.695
	[CENTRE = 3]	-.183	1.260	.021	1	.885	.833	.070	9.844
	[CENTRE = 4]	-2.534	1.056	5.764	1	.016	.079	.010	.628
	[CENTRE = 5]	-2.217	1.157	3.671	1	.055	.109	.011	1.052
	[CENTRE = 6]	0^b^	.	.	0	.	.	.	.
	EDSS	.228	.080	8.066	1	.005	1.256	1.073	1.470
	Age at onset	.047	.018	6.778	1	.009	1.049	1.012	1.087

The centre-defining variable (5 df) was forced to enter. Positive B values indicate a higher probability of CCSVI+.

**Figure 1:**
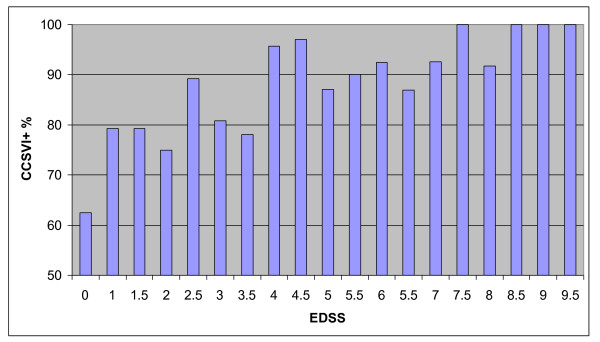
**Percentages of CCSVI-positive (CCSVI+) patients according to their EDSS score at the time of Doppler evaluation**.

Table 6 reports the mean values of EDSS and age at disease onset (the two variables selected by logistic regression) in patients with and in those without CCSVI from each centre. Overall, the frequency of CCSVI increased with increasing EDSS values (see Figure 1: chi-square for linear association 17.6, 1 df; p < 0.001).

**Table 6:** Mean values of EDSS and age at onset (/n) in CCSVI+ and CCSVI- patients in each MS centre.

MS centre	CCSVI	1	2	3	4	5	6	All centres
Age at onset	+	31/112	36/200	32/105	33/87	30/40	34/66	34/610
	
	-	28/34	31/9	28/11	28/31	29/9	31/4	29/98

p <		ns	ns	ns	0.01	ns	Ns	0.001

EDSS	+	3.6/112	5.1/200	4.3/105	4.4/87	6.0/40	4.6/66	4.5/610
	
	-	3.3/36	1.7/9	2.3/11	3.6/31	4.4/9	1.5/4	3.4/98

p <		ns	0.01	ns	ns	0.01	ns	0.001

The levels of significance are also reported.

Within-centre analyses revealed the following differences: MSSS was significantly higher in CCSVI-positive patients from Centres 2,5 and 6; age at disease onset was greater in CCSVI-positive subjects from Centre 4. With regard to the different types of MS, the following percentages of patients, in the whole sample, were found to be CCSVI-positive: relapsing-remitting (RR) 82%, secondary progressive (SP) 91%, primary progressive (PP) 92% (p < 0.001). With the exception of Centre 6, progressive patients (i.e. SP+PP) were more frequently CCSVI-positive, the difference reaching statistical significance in Centres 3 and 4 (a borderline significant difference, p = 0.055, was also found in Centre 2).

The associations between clinical variables and CCSVI were very high in the RR subgroup (see table 7); in the other two subgroups (SP and PP) no variable reached statistical significance, even though the differences in mean values tended, without exception, in the same direction as those recorded in the RR subgroup. We also performed a logistic regression analysis using a slightly different criterion to define the presence of CCSVI, i.e. fulfilment of at least three of the five CCSVI criteria instead of only two. Sixty-three percent of the patients could be diagnosed as CCSVI-positive using this cutoff. This model identified the same explanatory variables (age at disease onset and EDSS), fitted in a similar way.

**Table 7:** Mean values of clinical parameters in the RR subgroup.

	Age	Age at onset	Disease duration	EDSS	MSSS
CCSVI +	42	32	10	3.2	4.4

CCSVI -	39	27	11	2.5	3.2

p <	0.05	0.001	ns	0.001	0.001

The levels of significance are also reported.

## Discussion

In the limited literature available on this topic, CCSVI has been found in different proportions of MS subjects [20-22]. In the majority of studies, however, the frequency of CCSVI-positive patients exceeded that of CCSVI-positive healthy controls, suggesting that some link with MS (and possibly with other conditions) may exist.

However, the prevalence of CCSVI-positive patients varies greatly between centres, a fact confirmed by our data. Many factors may explain this variability in our study, including the use of different techniques, the relative unreliability of the test and the differences in the patient populations. The between-centre variability was found to be even higher when considering the single CCSVI criteria, although the third and first criteria tended to occur with greater frequency than the other three. Similar figures (even if with slightly lower percentages) were recently reported in the largest single center study by Zivadinov et al. [22]. We believe that the high frequency of the third criterion in MS patients is a particularly significant finding, since this criterion is related to direct visualisation of a venous anatomical abnormality, and should therefore be less operator-dependent. Although there were patients in whom flow abnormalities were seen in the absence of anatomical alterations, in these cases there is always a doubt that the B-mode anomalies were not seen. Since all the operators taking part in our study were trained in the method of Zamboni et al. in order to reduce inter-observer differences [23], the considerable variability in our data could be explained by the use of different equipment.

Differences in patient characteristics could account for another portion of the between-centre variability in our CCSVI findings. Indeed, higher frequencies of CCSVI were recorded in the centres which had the more severely affected patients. However, the relevance of this finding is not limited to the fact that it could partially explain the between-centre variability. Indeed, the association between CCSVI and EDSS (demonstrated by the logistic regression analysis) not only accounted for overall between-centre differences (possibly related to technical or other factors), but was also confirmed in the within-centre analyses, which showed consistently higher EDSS values in CCSVI-positive as opposed to CCSVI-negative subjects (the overall difference, corresponding to about one EDSS point, reached statistical significance in three centres). This finding, already reported by others [24], was reinforced in our study by the generally higher MSSS scores in CCSVI-positive patients.

The other variable consistently associated with CCSVI was age at disease onset, and this constituted another relevant finding: the CCSVI-positive patients were about five years older than the CCSVI-negative ones at disease onset. This association, to our knowledge never previously reported (Zivadinov et al. [22] reported only a slight difference in the frequency of CCSVI in a small group of paediatric MS), deserves particular attention. It could not be attributed only to differences in clinical course - PP subjects have generally a later disease onset and also more frequently present CCSVI - since it was also prominent in the RR subgroup. The association of older age at onset and a worse course in RR MS patients has been reported in a population-based study [25]. Moreover, a study on the clustering of multiple sclerosis recently highlighted the possible effect of 'exogenous' factors as determinants of a higher age at onset [26]. Indeed, also taking these observations into account, a putative model integrating 'autoimmune' and 'vascular' pathogenesis could fit our findings. In this framework, CCSVI could act as an 'exogenous' factor increasing mean age at onset by favouring the development of the disease in older subjects with a relatively low susceptibility to autoimmune diseases, some of whom would possibly otherwise never be ill. The recent finding that CCSVI is not linked to HLA DRB1*1501 status [27] confirms that venous insufficiency may act independently of the 'autoimmune trait'. On the contrary, these same findings are more difficult to integrate into an alternative model in which CCSVI is considered a secondary phenomenon.

Our data, while relatively robust due to the multicentre approach, need to be confirmed by replication and our hypothesis further evaluated. For instance, according to our model MS subjects without CCSVI should show a higher prevalence of MS-associated autoimmune disorders (i.e. thyroiditis or uveitis) and respond better to immunomodulating therapy.

Our data confirm that CCSVI is linked to MS, and tend to support the hypothesis that it could be a factor favouring disease development in relatively low-predisposed subjects and possibly a factor increasing disease severity. Every effort should be made to increase the reliability of the existing diagnostic techniques and to develop other ones, as the inter-centre differences observed in this and in other studies seem to be unacceptably high. The possible safety and efficacy of vascular intervention procedures should be assessed in blinded trials. Our data seem to suggest that the power of pilot interventional studies would be increased by selecting patients with a relatively older age at disease onset.

## Conclusion

This multicentre study confirms that a high proportion MS patients have CCSVI, as assessed by venous Doppler sonography. The relative high between-centre variability, particularly of single CCSVI criteria suggests that search for more reliable diagnostic procedures should be encouraged. The finding of significant relations between CCSVI and clinical aspects of MS testifies that CCSVI and MS are related entities. Nevertheless the significance of this relation is far from being assessed and further clinical, epidemiological as well as experimental research is needed.

### Limitations of Our Study

Our study has several limitations. First, is the lack of healthy controls (who were not available for each center); for this reason the main objective of the study was the correlation of the results with the clinical fenotypes of MS, and not the prevalence of CCSVI among the healthy and the MS population.

Second, the study was not blinded; although authors who performed the Doppler studies were not involved in the clinical management of the patients and were not aware of the clinical forms, the investigators knew that the subjects were MS patients.

However, we do not think the above limitations significantly compromise the validity of our findings.

## Competing interests

The authors declare that they have no competing interests.

## Authors' contributions

SB and RB equally contributed to conception and design, analysis and interpretation of data; drafting the manuscript, Critical revision of the paper, for important intellectual content. The first draft was written primarily by AR with some sections by GV, and extensively discussed, re-worked and edited by SB and RB. The statistical analysis was performed by AR. PC, MF, AG, MM, VB, SM and GB made substantial contributions to the study's conception and design. GV, ECT, EG, MA, LM, DM, CQ and AL contributed to acquisition of data. All authors read and approved the final manuscript.

## Pre-publication history

The pre-publication history for this paper can be accessed here:

http://www.biomedcentral.com/1471-2377/11/132/prepub
